# Impaired myocardial reserve underlies reduced exercise capacity and heart rate recovery in preterm-born young adults

**DOI:** 10.1093/ehjci/jeaa060

**Published:** 2020-04-17

**Authors:** Odaro J Huckstep, Holger Burchert, Wilby Williamson, Fernando Telles, Cheryl M J Tan, Mariane Bertagnolli, Linda Arnold, Afifah Mohamed, Kenny McCormick, Henner Hanssen, Paul Leeson, Adam J Lewandowski

**Affiliations:** 1 Oxford Cardiovascular Clinical Research Facility, Division of Cardiovascular Medicine, Radcliffe Department of Medicine, University of Oxford, Headley Way, John Radcliffe Hospital, Oxford OX39DU, UK; 2 Department of Biology, United States Air Force Academy, 2355 Faculty Drive, Suite 2P389, Colorado 80840, USA; 3 Hospital Sacré-Coeur Research Center, CIUSSS du Nord-de-l’Île-de-Montréal, 5400 Boulevard Gouin Ouest, Montreal, Quebec H4J1C5, Canada; 4 Department of Diagnostic Imaging and Radiotherapy, Facutly of Health Sciences, Universiti Kebangsaan Malaysia, Jalan Raja Muda Abdul Aziz, Kuala Lumpur 50300, Malaysia; 5 Department of Paediatrics, Headley Way, John Radcliffe Hospital, Oxford OX39DU, UK; 6 Department of Sport, Exercise and Health, University of Basel, Birsstrasse 320B, Basel 4052, Switzerland

**Keywords:** preterm birth, prematurity, exercise capacity, heart rate recovery, echocardiography, cardiac reserve

## Abstract

**Aims:**

We tested the hypothesis that the known reduction in myocardial functional reserve in preterm-born young adults is an independent predictor of exercise capacity (peak VO_2_) and heart rate recovery (HRR).

**Methods and results:**

We recruited 101 normotensive young adults (*n* = 47 born preterm; 32.8 ± 3.2 weeks’ gestation and *n* = 54 term-born controls). Peak VO_2_ was determined by cardiopulmonary exercise testing (CPET), and lung function assessed using spirometry. Percentage predicted values were then calculated. HRR was defined as the decrease from peak HR to 1 min (HRR_1_) and 2 min of recovery (HRR_2_). Four-chamber echocardiography views were acquired at rest and exercise at 40% and 60% of CPET peak power. Change in left ventricular ejection fraction from rest to each work intensity was calculated (EFΔ40% and EFΔ60%) to estimate myocardial functional reserve. Peak VO_2_ and per cent of predicted peak VO_2_ were lower in preterm-born young adults compared with controls (33.6 ± 8.6 vs. 40.1 ± 9.0 mL/kg/min, *P* = 0.003 and 94% ± 20% vs. 108% ± 25%, *P* = 0.001). HRR_1_ was similar between groups. HRR_2_ decreased less in preterm-born young adults compared with controls (−36 ± 13 vs. −43 ± 11 b.p.m., *P =* 0.039). In young adults born preterm, but not in controls, EFΔ40% and EFΔ60% correlated with per cent of predicted peak VO_2_ (*r*^2^ = 0.430, *P* = 0.015 and *r*^2^ = 0.345, *P* = 0.021). Similarly, EFΔ60% correlated with HRR_1_ and HRR_2_ only in those born preterm (*r*^2^ = 0.611, *P* = 0.002 and *r*^2^ = 0.663, *P* = 0.001).

**Conclusions:**

Impaired myocardial functional reserve underlies reductions in peak VO_2_ and HRR in young adults born moderately preterm. Peak VO_2_ and HRR may aid risk stratification and treatment monitoring in this population.

## Introduction

Prematurity is the leading cause of under-five child mortality,^1^ though more than 90% of those born preterm now survive into adulthood due to modern advances in pre- and post-natal care. Nevertheless, those born preterm suffer an increased prevalence of respiratory, cardiovascular, and neurological diseases in later life.^1^ Cardiovascular sequelae related to preterm birth include hypertension, early heart failure, and ischaemic heart disease, as well as increased mortality from cardiovascular disorders.^2–4^

The rate of maximum oxygen uptake during exercise (peak VO_2_) and heart rate recovery (HRR) are recognized as independent predictors of morbidity and all-cause mortality.^5^^,^^6^ Individuals born preterm have been reported to have lower peak VO_2_^7^ and have recently been shown to have impaired HRR,^8–10^ though the underlying mechanisms are unknown. Besides metabolic and cardiac alterations, interrupted pulmonary development, which normally continues late into the third trimester, is generally considered to contribute to potential exercise deficits.^7^ However, 80–85% of preterm births fall within the range of moderate to late prematurity (32–36 weeks gestational age) where acute neonatal respiratory distress syndrome is rarely seen.^11^ This variety of alterations, their association to gestational age, and the fact that impaired peak VO_2_ and HRR are seen in a range of conditions^12^ limits risk stratification and compromises the interpretation of impaired peak VO_2_ and HRR in individuals with a history of preterm birth.

Our previous investigations identified an altered cardiac phenotype in preterm-born young adults, even in those born moderate to late preterm.^13^^,^^14^ These cardiac alterations include an impaired increase in left ventricular (LV) ejection fraction (EF) between rest and light to moderate exercise intensity, suggestive of a reduced myocardial functional reserve.^15^ We therefore tested the hypothesis that this reduction in myocardial functional reserve in preterm-born young adults is an independent predictor of peak VO_2_ and HRR.

## Methods

### Study design

We completed the Young Adult Cardiovascular Health sTudy (YACHT); an observational, case-control study with a primary outcome measure of cardiopulmonary exercise capacity to investigate cardiovascular structure and function, along with cardiopulmonary capacity and exercise stress response in preterm- and term-born young adults. Participants completed a detailed multimodal set of study measures including 24-h ambulatory blood pressure monitoring, cardiopulmonary exercise testing (CPET), and cardiac imaging. Ethical approval for YACHT was granted by the South Central Berkshire Research Ethics Committee (14/SC/0275). Study registration was completed via www.clinicaltrials.gov (NCT02103231).

### Study population

A total of 149 participants aged 18–40 years were recruited through purposive active and passive recruitment as previously described.^15^ In total, 101 non-hypertensive young adult participants (47 preterm-born, 54 term-born) meeting the inclusion and exclusion criteria were included in this analysis.^15^ Cohort characteristics are provided in *Table 1*.

**Table 1:** Cohort clinical characteristics

Group characteristics	Preterm-born young adults (*n* = 47)	Term-born young adults (*n* = 54)	*P*-value
Demographics and anthropometrics			
Age (years)	22.7 ± 3.04	23.6 ± 3.8	0.239
Gestational age (weeks)	32.8 ± 3.23	39.5 ± 1.37	**<0.001**
32–36 weeks, *n* (%)	38 (80.9)	0 (0.0)	
28–31 weeks, *n* (%)	5 (10.6)	0 (0.0)	
<28 weeks, *n* (%)	4 (8.5)	0 (0.0)	
Male, *n* (%)	14 (30)	26 (48)	0.061
Birth weight (grams)	1916 ± 806	3390 ± 424	**<0.001**
Gestational hypertension, *n* (%)	8 (17.0)	0 (0.0)	**0.002**
Small for gestational age, *n* (%)	2 (4.3)	0 (0.0)	0.214
Height (cm)	167 ± 9	175 ± 10	**<0.001**
BMI (kg/m^2^)	23.3 ± 4.5	22.7 ± 2.7	0.401
Biochemistry			
Total cholesterol (mmol/L)	4.72 ± 0.65	4.18 ± 0.77	**0.001**
HDL (mmol/L)	1.49 ± 0.31	1.47 ± 0.26	0.882
LDL (mmol/L)	2.80 ± 0.71	2.32 ± 0.60	**0.001**
Triglycerides (mmol/L)	1.12 ± 0.66	0.87 ± 0.36	**0.031**
High sensitivity CRP (mg/L)	1.57 ± 2.42	1.14 ± 1.96	0.412
Glucose (mmol/L)	5.02 ± 0.41	4.82 ± 0.51	**0.030**
Insulin (pmol/L)	51.1 ± 29.0	35.8 ± 29.4	**0.012**
Insulin resistance	0.96 ± 0.54	0.68 ± 0.59	**0.020**
Brachial blood pressure (mmHg)			
Resting systolic	119 ± 9	115 ± 8	**0.014**
Resting diastolic	70 ± 8	66 ± 5	**0.014**
Average 24h systolic	119 ± 6	119 ± 8	0.385
Average 24h diastolic	71 ± 5	69 ± 5	0.065
Cardiac function			
Heart rate (b.p.m.)	75 ± 13	61 ± 12	**<0.001**
Ejection fraction (%)	63.1 ± 5.4	63.4 ± 4.7	0.624
Stroke index (mL/m^2^)	29.6 ± 6.4	32.2 ± 7.8	0.173
Pulmonary function			
FVC (% of predicted)	109 ± 15.2	109 ± 12.0	0.917
FEV_1_/FVC (%)	81.4 ± 6.4	81.6 ± 8.1	0.795

Group characteristics presented as mean ± SD. Biochemistry, blood pressure, cardiac function, and pulmonary function *P*-values adjusted for sex. Insulin resistance was calculated using the Homeostatic Model Assessment Index (HOMA) calculator (www.dtu.ox.ac.uk/homacalculator/). Bold *P* values are statistically significant (*P*<0.05).

BMI, body mass index; FEV_1_, forced expiratory volume in 1 s; FVC, forced vital capacity; HDL, high density lipoprotein; LDL, low-density lipoprotein.

### Study visit

Participants were instructed to fast overnight for 12 h but to drink plenty of water prior to attending a study visit at the University of Oxford Centre for Clinical Magnetic Resonance (OCMR) and Oxford Cardiovascular Clinical Research Facility (CCRF) in the John Radcliffe Hospital in Oxford, UK. All measurements were completed by trained study investigators and clinical staff, including height, weight, venepuncture, and blood pressure as previously described (see Supplementary material online).^15^

#### Spirometry

Spirometry was performed according to standard guidelines by using the Cortex Metalyzer 3B (Metalyzer 3B, Cortex Biophysik GmbH, Leipzig, Germany).^16^ The spirometer underwent calibration checks of the flow and volume before each test using a Futuremed’s 3.0-L calibration syringe (Futuremed, CA, USA). The participants were seated and guided through the test wearing the nose-clips with the lips tightly sealed around the mouthpiece. A minimum of three acceptable forced expiratory manoeuvres were made for every participant. To ensure consistency and high quality of the spirometry data, visual checks of the waveforms were done before the highest values from the data were selected and recorded on a clinical research form. The parameters recorded included forced expiratory volume in 1 s (FEV_1_) and forced vital capacity (FVC), as well as per cent of predicted values according to Quanjer *et al.*^17^

#### CPET and HRR

Following spirometry, participants completed a peak CPET on a seated stationary cycle ergometer (Ergoline GmbH, Bitz, Germany) using a validated incremental protocol with respiratory gases collected and measured using the same Metalyzer as used during spirometry. Heart rate was recorded using continuous electrocardiogram (ECG) monitoring and blood pressure was recorded every 4 min using a manual mercury sphygmomanometer (ACCOSON Freestyle, Essex, UK). Participants maintained a rate of 60 revolutions per minute during the test, which began with one quiescent minute of resting measurements followed by a 2-min warm-up with a 20 watt workload. After the warm-up period, workload increased to 35 watts. To normalize test duration to ∼8–12 min, participants who reported higher activity or fitness levels had their workload increased to 75 watts after the warm-up period. Workload then incremented by 15 watts each minute and participants cycled continuously until exhaustion prevented them from maintaining at least 50 revolutions per minute or safety termination criteria were met.^18^ Participants then completed a 2-min cool-down period at 50 watts and the revolutions per minute of their preference. Per cent of predicted peak VO_2_ was calculated based on the Wassermann weight algorithm.^19^

#### Stress echocardiography

After completing CPET, participants had a 30-min recovery period during which they were offered water and a cereal bar. Sub-maximal exercise stress echocardiography was then performed on study subjects for 3 min at 40% and 60% of their peak exercise wattage achieved during CPET testing as previously described.^15^ In brief, after 2 min at each prescribed workload and with steady-state heart rate verified, apical four-chamber views optimized for the LV and pulsed wave Doppler imaging of mitral inflow were collected at each stage using either a Philips iE33 or Philips EPIQ 7C (Philips Healthcare, Surrey, UK) cardiology ultrasound machine with an X5-1 xMATRIX array transducer to compare with resting echocardiography measures as previously described.^15^ Participants were given a 3-min rest period between each stage. Offline image analysis was only performed on images where heart rate was within 5% of maximum achieved steady-state heart rate for a given stage.

### Data processing

#### Echocardiography analysis

Echocardiography analysis was completed offline in accordance with standard guidelines as previously described (see Supplementary material online, Methods).^15^ Resting LVEF was calculated via the bi-plane Modified Simpson’s method using apical four-chamber and apical two-chamber views while sub-maximal exercise LVEF at 40% and 60% were calculated by the single-plane Modified Simpson’s method using apical four-chamber views. EF change was calculated from rest to 40% and 60% exercise intensity (EFΔ40% and EFΔ60%). Intraclass correlation coefficients for LV ejection fraction at rest and under physiological stress at 40% and 60% for intra/inter-observer variability were 0.89/0.85, 0.90/0.89, and 0.89/0.90, respectively.

#### Heart rate recovery

The ECG of each participant after the cessation of exercise was inspected by two researchers for valid readings at 1 and 2 min after exercise. Heart rate was smoothed within the Metalyzer Software (MetaSoft Studio, Cortex Biophysik GmbH, Leipzig, Germany) using the moving average (time interval) set to 15 s so that an average from the data points in this specified time interval (around the supporting point) was formed. The heart rates at 1 and 2 min after the cessation of exercise were then identified and extracted for each participant. HRR was defined as the absolute drop in heart rate from peak heart rate to 1 min (HRR_1_) and 2 min after exercise (HRR_2_).

### Statistical analysis

Statistical analysis was performed using SPSS Version 25. Shapiro–Wilk testing and visual inspection were used to assess normality of variable distribution. Direct, between-group comparisons were performed using independent-samples Student’s *t*-tests for normally distributed data and Mann–Whitney and Kruskal–Wallis tests for non-normally distributed data. Between-group comparisons were adjusted for sex. Linear regression modelling was completed using forced entry with missing data excluded pairwise. Our final study numbers *n* = 47 vs. *n* = 54 allowed us to detect a 0.77-standard deviation difference between groups for study measures, powered at 80% with *P *=* *0.05. *P*-values <0.05 were considered statistically significant.

## Results

### Clinical characteristics

There were no differences in body mass index or age, but between-group sex distribution approached significance with a 30% male preterm group vs. a 48% male term-born group, *P *=* *0.061. Between-group comparisons were, therefore, adjusted for sex throughout. Resting LVEF and stroke index measured by echocardiography were similar between groups. Baseline pulmonary function measured by spirometry was also similar between groups with no significant differences in per cent of predicted FVC or FEV_1_/FVC. Baseline group characteristics are provided in *Table 1*.

### Exercise characteristics

#### Reduced aerobic exercise capacity and HRR in preterm-born young adults

**Figure 1:**
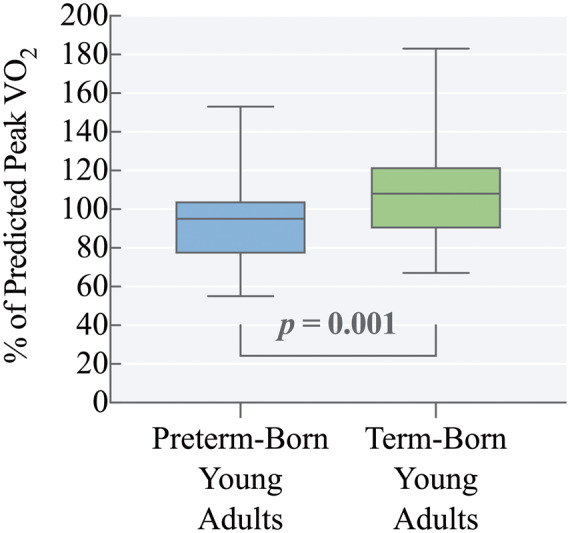
Reduced rate of maximum oxygen consumption during exercise in preterm-born young adults. Preterm-born young adults achieved 94% ± 20% (*n *=* *47) of their predicted peak VO_2_ utilization rate vs. 108% ± 25% (*n *=* *54) in term-born controls, *P *=* *0.001. Boxplots represent min to max.

**Figure 2:**
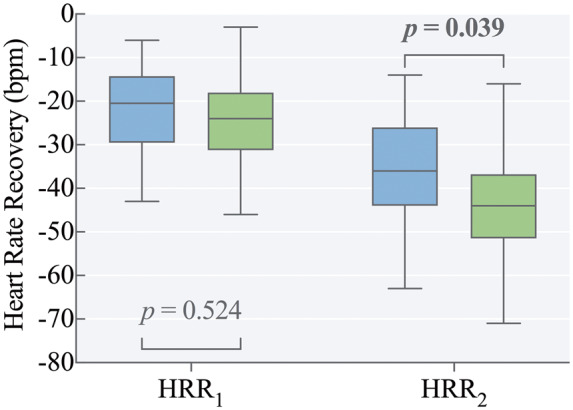
Heart rate recovery at one and two minutes post exercise. There was no significant difference in heart rate recovery after one minute (HRR1) between preterm-born young adults in blue (*n* = 40) and term-born controls in green (*n* = 46). Heart rate recovery after two minutes (HRR2) was significantly reduced in preterm-born young adults (*n* = 39) compared to term-born controls (*n* = 46). Boxplots represent min to max.

Peak cardiopulmonary exercise capacity was lower in preterm-born young adults, who on average, achieved 94% ± 20% of their predicted peak VO_2_ vs. 108% ± 25% in term-born controls, *P *=* *0.001 (*Figure 1*). Peak VO_2_ measured in mL/kg/min was also lower in those born preterm (33.6 ± 8.6 vs. 40.1 ± 9.0 mL/kg/min, *P *=* *0.003). Heart rate at the ventilatory anaerobic threshold (VAT) was higher in the preterm group (132 ± 18 vs. 124 ± 17 beats/min, *P *=* *0.038) but peak achieved heart rate was similar between groups. Ventilatory reserve at peak ventilation rate was similar between preterm-born and term-born individuals (23.3% ± 17.8% vs. 21.2% ± 18.3% of maximum minute ventilatory volume, respectively, *P *=* *0.513) (*Table 2*). As shown in *Figure 2*, HRR_1_was similar between groups (-21 ± 9 vs. -24 ± 9 b.p.m., *P *=* *0.457) and remained similar after additional adjustment for per cent of predicted peak VO_2_ (*P *=* *0.492). HRR_2_ was slower in preterm-born young adults (-36 ± 13 vs. -43 ± 11 b.p.m., *P = *0.039), but no significant difference was found after additional adjustment for per cent of predicted peak VO_2_ (*P *=* *0.813). Exercise characteristics are presented in *Table 2*.

**Table 2:** Exercise characteristics

	Preterm-born young adults (*n* = 47)	Term-born young adults (*n* = 54)	*P*-value
Exercise performance			
Per cent of predicted peak VO_2_	94 ± 20	108 ± 25	**0.001**
Peak VO_2_ (mL/kg/min)	33.6 ± 8.6	40.1 ± 9.0	**0.003**
OUE slope	2.39 ± 0.73	3.05 ± 0.89	**0.001**
VE/VCO2 slope	28.1 ± 3.3	25.9 ± 3.8	**0.011**
Peak RER	1.19 ± 0.06	1.19 ± 0.06	0.738
Cardiovascular function			
Heart rate at VAT (b.p.m.)	132 ± 18	124 ± 17	**0.038**
Heart rate at 60% intensity (b.p.m.)	159 ± 11	151 ± 14	0.055
Peak HR achieved (b.p.m.)	189 ± 9	186 ± 11	0.218
HRR_1_ (b.p.m.)	−21 ± 9	−24 ± 9	0.457
HRR_2_ (b.p.m.)	−36 ± 13	−43 ± 11	**0.039**
Systolic blood pressure at 60% intensity (mmHg)	146 ± 16	149 ± 14	0.567
Diastolic blood pressure at 60% intensity (mmHg)	81 ± 10	77 ± 7	0.139
Peak systolic blood pressure (mmHg)	165 ± 22	167 ± 21	0.885
Peak diastolic blood pressure (mmHg)	89 ± 11	82 ± 10	**0.002**
Peak pulse pressure (mmHg)	76 ± 23	86 ± 23	0.168
Pulmonary function			
Breaths per minute at peak VO_2_	44.3 ± 6.7	47.4 ± 11.8	0.188
Ventilatory reserve at peak (%)	23.3 ± 17.8	21.2 ± 18.3	0.513
Objective measure of physical activity			
MVPA (h/week)	14.2 ± 5.8	14.9 ± 6.1	0.633
VPA (h/week)	0.9 ± 0.9	1.2 ± 1.2	0.146

Exercise characteristics presented as mean ± SD. *P*-values adjusted for sex. Bold *P* values are statistically significant (*P*<0.05).

HRR, heart rate recovery; MVPA, moderate to vigorous physical activity; OUE Slope, oxygen uptake efficiency slope; RER, respiratory exchange ratio; VAT, ventilatory anaerobic threshold; VE/VCO2, minute ventilation/carbon dioxide production;VO_2_, oxygen uptake; VPA, vigorous physical activity.

#### Change in EF during exercise predicts exercise capacity and HRR in preterm-born young adults

**Figure 3:**
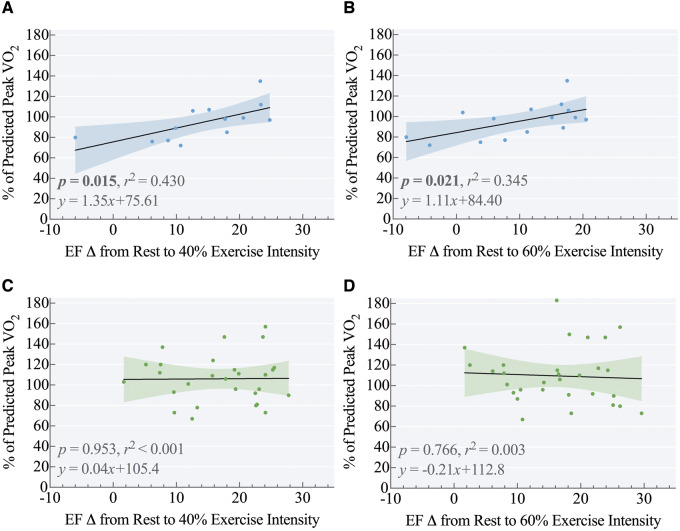
Increase in LVEF from baseline to 40% and 60% as a predictor of peak VO_2_. Change in LVEF from rest to 40% exercise intensity (EFΔ40%) was predictive of percent of predicted peak VO_2_ in the preterm-born group (Panel A: r^2^ = 0.430, *P* = 0.015, *n* = 13), but not in full-term born controls (Panel C: r^2^ < 0.001, *P* = 0.953, *n* = 28). Change in LVEF from rest to 60% exercise intensity (EFΔ60%) was similarly predictive of percent of predicted peak VO_2_ in the preterm-born group (Panel B: r^2^ = 0.345, *P* = 0.021, *n* = 15), but not in full-term controls (Panel D: r^2^ = 0.003, *P* = 0.766, *n* = 30).

**Figure 4:**
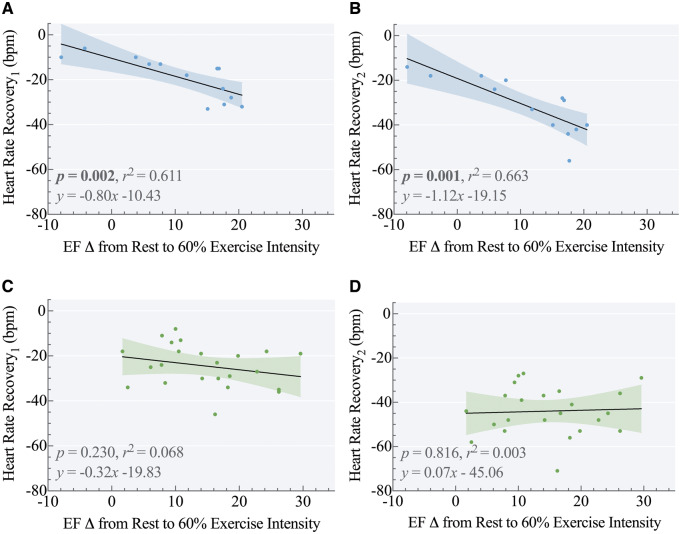
Change in LVEF from rest to 60% exercise intensity as a predictor of heart rate recovery. Change in LVEF from rest to 60% exercise intensity (EFΔ60%) was predictive of heart rate recovery at 1 and 2 min (HRR_1_ and HRR_2_, respectively) post-exercise in preterm-born young adults (Panel *A*: *r*^2^ = 0.611, *P *=* *0.002, *n *=* *13 and Panel *B*: *r*^2^ = 0.663, *P *=* *0.001, *n *=* *13, respectively) but not in term-born young adults (Panel *C*: *r*^2^ = 0.068, *P *=* *0.230, *n *=* *23 and Panel *D*: *r*^2^ = 0.003, *P *=* *0.816, *n *=* *23, respectively).

Bivariate correlations were carried out to investigate whether EFΔ, pulmonary, and physical activity measures were associated with per cent of predicted peak VO_2_ and HRR. As shown in Supplementary data online, *Table S1*, EFΔ40% as well as EFΔ60% were correlated with per cent of predicted peak VO_2_ only in preterm-born young adults (*r*^2^ = 0.430, *P *=* *0.015 and *r*^2^ = 0.345, *P *=* *0.021, respectively) (*Figure 3A and C*). EFΔ60% was correlated with HRR_1_ and HRR_2_ only in preterm-born young adults (*r*^2^ = 0.611, *P *=* *0.002 and *r*^2^ = 0.663, *P *=* *0.001, respectively) (*Figure 4A and C*).

#### Multivariable analysis for confounding effects of pulmonary capacity and dynamic function in preterm-born subjects

To determine if the relationships between EFΔ and per cent of predicted peak VO_2_ as well as HRR in preterm-born subjects were significantly influenced by pulmonary volume or dynamic pulmonary function, we completed multivariable linear regression analyses. Based on bivariable regression analyses, significant variables were carried forward to a multivariable model. To predict per cent of predicted peak VO_2_ in the preterm group, we included per cent predicted FVC, ventilatory reserve at peak VO_2_, and EFΔ40% and then EF60Δ% in two separate models. EFΔ40% and EFΔ60% remained the strongest predictors of per cent of predicted peak VO_2_ (beta = 0.538, *P *=* *0.084 and beta = 0.506, *P *=* *0.152) (*Table 3*). For the prediction of HRR_1_ and HRR_2_ in the preterm group, we included per cent of predicted FVC and EFΔ60% in the model. EFΔ60% remained the strongest predictor of HRR_1_ (beta = -1.030, *P *=* *0.002) and HRR_2_ (beta = -0.948, *P *=* *0.003).

**Table 3:** Multiple linear regression models of per cent of predicted peak VO_2_, HRR_1_, and HRR_2_ in preterm-born young adults

	Preterm-born young adults (*n* = 47)			
Per cent of predicted peak VO_2_	*B*	95%CI	Beta	*P*-value
Per cent of predicted FVC	0.176	−0.634 to 0.983	0.132	0.634
Vent. reserve (%)	−0.234	−0.883 to 0.415	−0.205	0.435
EFΔ40% (%)	1.269	−0.208 to 2.746	0.538	0.084
Per cent of predicted peak VO_2_				
Per cent of predicted FVC	0.031	−0.905 to 0.967	0.023	0.943
Vent. reserve (%)	−0.270	−0.886 to 0.345	−0.236	0.354
EFΔ60% (%)	1.162	−0.498 to 2.823	0.506	0.152
HRR_1_ (b.p.m.)				
Per cent of predicted FVC	0.227	−0.106 to 0.561	0.367	0.159
EFΔ60% (%)	−1.099	−1.673 to −0.525	−1.030	**0.002**
HRR_2_ (b.p.m.)				
Per cent of predicted FVC	0.170	−0.293 to 0.633	0.198	0.432
EFΔ60% (%)	−1.405	−2.203 to −0.608	−0.948	**0.003**

Bold *P* values are statistically significant (*P*<0.05). FVC represents forced vital capacity; EFΔ40%, ejection fraction change from rest to 40% exercise intensity; EFΔ60%, ejection fraction change from baseline to 60% exercise intensity; *B*, unstandardized regression coefficient.

## Discussion

This study reports for the first time that normotensive, moderately preterm-born young adults exhibited reduced aerobic exercise capacity and slower HRR. These reductions were strongly related to their level of impairment in LV systolic function when going from rest to mild and moderate levels of exercise intensity. In addition, mechanical pulmonary function in this moderately preterm group was similar to controls, and exercise LV systolic response remained the strongest predictor of exercise capacity and HRR in preterm-born young adults after adjusting for pulmonary volume and dynamic function.

### Impaired preterm myocardial functional reserve underlies reduced exercise capacity

Several diseases including cardiac amyloidosis,^20^ diabetes,^21^ metabolic syndrome,^22^ and heart failure of various aetiologies^23^^,^^24^ are associated with impaired cardiac contractility during exercise with overall exercise capacity subsequently reduced. However, the mechanisms responsible for diminished increases in exercise EF are understood to vary in each instance, including poor coronary blood flow resulting in challenged sub-endocardial perfusion^20^ and dysfunctional myocardial energetics with reduced adenosine triphosphate production.^21^^,^^22^ Due to the structural remodelling with thicker myocardial walls in the LV in those born preterm,^13^ they may have greater susceptibility to subtle, subclinical deficits in coronary blood flow, though this would require further study to assess. In this study, however, we did observe elevated low-density lipoprotein cholesterol, triglycerides, plasma glucose, plasma insulin, and insulin resistance in the preterm group compared with controls.^15^ Other previous studies of preterm-born individuals have reported similar findings of impaired glucose and lipid metabolism in varied preterm-born study groups.^13^^,^^25^ Accordingly, while myocardial energetics were not assessed in this work, the presence of subclinical alterations in glucose and lipid metabolism suggests that research into the role of myocardial energetics as a contributing factor to the impaired systolic response to exercise in preterm-born subjects is warranted.

### Pulmonary physiology does not explain reduced preterm exercise capacity

We observed that impaired LV systolic response to exercise stress was associated with diminished exercise capacity in normotensive preterm-born young adults. However, because lung development continues into the third trimester, pulmonary function is often considered a risk area for deficits and vulnerability in preterm-born infants. In particular, the saccular and alveolar phases of development may be interrupted by premature delivery. In spite of this, spirometric values in our preterm-born group were similar to controls. Interestingly, this comports with a growing body of evidence that moderate to late preterm birth may be less impacting of pulmonary capacity later in life than once thought.^26^

In this study, the similar pulmonary volume and dynamic function between study groups and results from the multivariable analysis indicate that change in EF from rest to exercise, rather than mechanical ventilation, predicted exercise capacity. The elevated VE/VCO_2_ slope in the preterm group indicates that exercise was limited by cardiac function. Such elevations most commonly occur secondary to deficits in cardiac performance, which drive a compensatory response of increased ventilation.^27^ This ventilation perfusion mismatch is common in heart failure, where exercise VE/VCO_2_ slopes often exceed a value of 30. In the otherwise healthy, preterm-born young adults in this study, the presence of this compensatory increased ventilatory response relative to CO_2_ production and O_2_ uptake, combined with typical ventilatory reserve margin (23.3%) are consistent with cardiac limited exercise performance.^27^

### Systolic myocardial impairment and delayed heart rate recovery

In this article, we have shown that HRR is reduced at 2 min after exercise in normotensive, moderately preterm-born young adults and that this associated with impaired systolic function during exercise rather than pulmonary function. The metaboreflex is well known for its effect on blood pressure but the effect on post-exercise heart rate remains controversial.^28^ However, it seems plausible that systolic myocardial impairment in the preterm-born young adults results in hypoperfusion of metabolite eliminating organs, and thus the clearance and deactivation of the metaboreflex are delayed. Furthermore, delayed HRR is commonly seen in heart failure populations^6^ and metaboreflex modulation plays a key role in the ‘muscle hypothesis’.^29^ As Crisafulli^30^ explains, the normal haemodynamic response to metaboreflex activation is, among other factors, an increase in arterial blood pressure achieved by increased cardiac contractility boosting stroke volume to maintain adequate muscle perfusion. In absence of normal contractile reserves, exaggerated peripheral vasoconstriction is required to sustain pressure, which increases systemic vascular resistance.^30^ The increased afterload further impairs stroke volume which exacerbates muscle perfusion deficits and ultimately produces early fatigue. Whether such a mechanism exists in conjunction with preterm birth would require detailed future research. However, Crump *et al.*^3^ recently reported an association of preterm birth with ischaemic heart disease even in those born moderately preterm, which further suggests this mechanism might exist.

To our knowledge, apart from one study comparing HRR after sub-maximal exercise,^10^ only two other studies investigated HRR after peak exercise testing. The first was conducted on healthy very preterm-born adolescents^9^ and the second on healthy very preterm-born young adults.^8^ In contrast to our study, they found reductions in HRR after the first minute. Furthermore, HRR remained lower in the very preterm young adults of the second study after adjusting for maximal oxygen consumption to control for fitness level, which was not the case for the moderately preterm-born individuals in our study. Whether this discrepancy indicates more severe or additional impairments in more premature populations remains to be determined as methodological differences (active vs. passive recovery) hinder direct comparisons.^31^ Interestingly, the mentioned second study also found that hypoxia did not further slow-down HRR in very preterm young adult individuals compared with controls. This result may support the muscle hypothesis, as compensatory increases in stroke volume or vasoconstriction, to counter the oxygen drop, may already be working at their physiological limits to compensate for the reduced contractile reserve. Although HRR is widely used as a prognostic marker,^6^ the underlying autonomic mechanisms, especially in preterm populations, are incompletely understood, and thus further research is needed to elucidate autonomic function contributions to differences in HRR.

### Clinical implications

Overall, the moderately premature gestational age of the preterm-born group makes our results more broadly relevant, given that this reflects the demographic of the majority of preterm births.^32^ The reduced association between physical activity and exercise capacity in the preterm group suggests that prematurity may alter cardiovascular adaptation to aerobic training and require tailored approaches to lifestyle intervention, though randomized control trials will be needed to assess this. The finding that a lower myocardial functional reserve seen in those young adults born preterm explains a significant proportion of the lower peak VO_2_ and slower HRR adds further clinical relevance to the altered preterm cardiac phenotype. In addition, peak VO_2_ and HRR in this population may be important and beneficial for future risk stratification and treatment monitoring, acting as surrogate measures of reduced myocardial functional reserve. Continued investigation and longitudinal follow-up will be necessary to more fully understand the clinical implications of these findings.

### Study limitations

We designed YACHT as an observational study around a single, full-day study visit. As the exercise stress testing came towards the end of the visit in order not to confound other measures, not all individuals were willing to continue with the sub-maximal exercise testing and echocardiography stress testing component. Also, due to challenges with imaging quality, not all echocardiography scans were of sufficient quality for analysis. Finally, the overall sample size was modest with a lower percentage of males in the preterm cohort, and therefore, analyses were adjusted for sex where appropriate. Nevertheless, the dataset was sufficiently powered to make between-group comparisons for the YACHT primary outcome of cardiopulmonary aerobic fitness as well as LVEF at rest, 40%, and 60% of maximal exercise capacity using echocardiography.

There was a wide gestational age range in our participants with the majority of our preterm-born individuals born moderate to late preterm, which reflects the demographic of the general preterm population.^32^ Although this makes our findings more relevant to a larger proportion of the population, larger studies will be needed to fully explore to what extent the severity of the LV systolic response to exercise is altered specifically in very and extreme preterm-born adults, who are at greater risk of perinatal complications. Finally, while pulmonary function and objective measurement of physical activity (accelerometry) were included in this analysis, direct assessment of pulmonary gas diffusion and broader ranging lifestyle characteristics, including historical physical activity profile, were not assessed.

## Conclusions

Preterm-born young adults have reduced exercise capacity and HRR, which relate to the preterm functional phenotype of reduced LV systolic response to physical exercise. Although below average, exercise capacity was within the normal range and did not reflect acute clinical deficits. Further research is needed to develop a more complete understanding of cardiopulmonary exercise capacity and function in the preterm-born population, as well as which mechanisms underlie reduced peak oxygen consumption and HRR in preterm individuals of varying degrees of prematurity.

## Supplementary data

Supplementary data are available at *European Heart Journal - Cardiovascular Imaging* online. 

## Supplementary Material

jeaa060_Supplementary_Data
