# Erratum to: Impaired myocardial reserve underlies reduced exercise capacity and heart rate recovery in preterm-born young adults

**DOI:** 10.1093/ehjci/jeaa116

**Published:** 2020-04-24

[*Eur Heart J – Cardiovasc Imaging* 2020; doi:10.1093/ehjci/jeaa060]

In the original article, the abstract was accidentally omitted. This has now been added in. The Publisher apologizes for the error. In addition, Tables 1, 2 and 3 had some missing units and these have now been added.

