# Supplementary material for: Impaired myocardial reserve underlies reduced exercise capacity and heart rate recovery in preterm-born young adults
Source: Eur Heart J Cardiovasc Imaging. 2020 Apr 17;22(5):572–80. doi: 10.1093/ehjci/jeaa060 (PMC8081423; doi:10.1093/ehjci/jeaa060)
Supplement: jeaa060_Supplementary_Data [file jeaa060_supplementary_data.docx]

**Title:** *Impaired Myocardial Reserve Underlies Reduced Exercise Capacity and Heart Rate Recovery in Preterm-Born Young Adults*

**Short Title:** *Exercise Capacity in Preterm-Born Adults*

**SUPPLEMENTAL MATERIAL**

***Authors:*** ^a,b*^Odaro J Huckstep, DPhil; ^a*^Holger Burchert, MSc; ^a^Wilby Williamson, MBBS DPhil; ^a^Fernando Telles, MD; ^a^Cheryl MJ Tan, MRes; ^a,c^Mariane Bertagnolli, PhD; ^a^Linda Arnold, MSc; ^a,d^Afifah Mohamed, MSc; ^e^Kenny McCormick MBBCh; ^f^Henner Hanssen, MD; ^a^Paul Leeson, PhD FRCP; ^a++^Adam J Lewandowski, DPhil

***^a^Oxford Cardiovascular Clinical Research Facility, Division of Cardiovascular Medicine, Radcliffe Department of Medicine, University of Oxford, Oxford, UK***

***^b^Department of Biology, United States Air Force Academy, USA***

***^c^Hospital Sacré-Coeur Research Center, CIUSSS du Nord-de-l’Île-de-Montréal, Canada***

***^d^Facutly of Health Sciences, Universiti Kebangsaan Malaysia, Kuala Lumpur, Malaysia***

***^e^Department of Paediatrics, University of Oxford, Oxford, UK***

***^f^Department of Sport, Exercise and Health, University of Basel, Basel, Switzerland***

*These authors contributed equally to this work

***++Corresponding Author:***

*Dr Adam Lewandowski, Oxford Cardiovascular Clinical Research Facility, Division of Cardiovascular Medicine, Radcliffe Department of Medicine, University of Oxford. John Radcliffe Hospital, Oxford, UK OX39DU.*

*Email: adam.lewandowski@cardiov.ox.ac.uk*

*Tel: +44(0)1865223680*

*Fax: +44(0)1865572840*

**STUDY MEASURES**

Anthropometrics

Height was measured to the nearest centimetre and weight measured to the nearest 0.1 kg with footwear removed and participants wearing light clothing using an integrated height and weight measurement station (Seca, Birmingham, United Kingdom).

Blood Pressure Measurements

After a 5-min acclimation period, 3 resting peripheral blood pressure measurements were recorded using a digital blood pressure monitor (GE Dinamap V100, GE Healthcare, Chalfont St. Giles, United Kingdom) on the left arm with the last 2 readings averaged and subsequently analyzed. Twenty-four-hour ambulatory blood pressure monitoring was initiated at the end of the study visit using oscillometric, ambulatory devices (TM-2430, A&D Instruments, Abingdon, United Kingdom). Correct cuff size was chosen based on arm circumference. Subjects were instructed to remain still during measurements. Measurements were automatically taken every 30 min during daytime and then hourly from 11:00 pm to 7:00 am. Subjects completed a diary documenting hours asleep and awake.

Blood Sampling

Water was offered and available to participants throughout their study visit to ensure euhydration. After collecting fasting, at-rest blood samples from the antecubital fossa by venepuncture or indwelling venous catheter, participants were provided a standardised snack of two cereal bars. Samples were centrifuged within 15 minutes of collection. Separated plasma and serum were then pipetted and stored at -80ºC for future analysis. Fasting blood biochemistry was measured at the Oxford John Radcliffe Hospital Biochemistry Laboratory through the use of routine validated assays with clinical level quality controls. Insulin Resistance was calculated using the Homeostasis Model Assessment (HOMA) calculator ([www.dtu.ox.ac.uk/homacalculator/](http://www.dtu.ox.ac.uk/homacalculator/)).

Echocardiography Analysis

The Philips Xcelera 3.3 (Philips Healthcare Informatics, Belfast, Northern Ireland) and TomTec Image Arena 4.6 (Chicago, IL, United States) software suites were utilised for image analysis. End-diastole was set at the point of mitral valve closure and end systole was set at the point of minimum LV cavity size, LV endocardial borders were manually contoured at end-diastole and end-systole to allow calculation of end-diastolic volume (EDV) and end-systolic volume (ESV).

Fitting Monitoring Devices

24-hour ambulatory blood pressure monitoring was initiated at the end of the study visit using oscillometric, ambulatory devices (TM-2430, A&D Instruments, Abingdon, United Kingdom). Correct cuff size was chosen based on arm circumference. Subjects were instructed to remain still during measurements. Measurements were automatically taken every 30 minutes during daytime and then hourly from 11:00 PM to 7:00AM. Subjects completed a diary documenting asleep and awake hours. Additionally, participants received an atriaxial accelerometer (AX3 Axivity, Newcastle, UK) after the visit to complete seven days of objectively measured physical activity. Moderate to vigorous physical activits (MVPA) and vigorous physical activity (VPA) were derived from the data using previously described methods (1).

Supplemental Table 1. Bivariate Correlations of Percent of Predicted Peak VO_2_, HRR_1_ and HRR_2_

|  | **Preterm Born Young Adults**  **n = (47)** | | **Full-term Born Young Adults**  **n = (54)** | |
| --- | --- | --- | --- | --- |
| **Correlates of % of Predicted Peak VO_2_** | ***r*** | ***p*-value** | ***r*** | ***p*-value** |
| FEV_1_/FVC (%) | -0.190 | 0.212 | 0.091 | 0.527 |
| Percent of predicted FVC | 0.400 | **0.006** | 0.216 | 0.128 |
| Vent. Reserve | -0.380 | **0.010** | -0.500 | **<0.000** |
| EFΔ40% | 0.655 | **0.015** | 0.012 | 0.953 |
| EFΔ60% | 0.587 | **0.021** | -0.057 | 0.766 |
| MVPA (hours/week) | 0.113 | 0.451 | 0.264 | 0.067 |
| VPA (hours/week) | 0.191 | 0.199 | 0.430 | **0.002** |
| **Correlates of HRR_1_** |  |  |  |  |
| FEV_1_/FVC (%) | -0.094 | 0.566 | 0.117 | 0.437 |
| % of predicted FVC | -0.329 | **0.038** | -0.100 | 0.511 |
| Vent. Reserve | 0.167 | 0.304 | 0.311 | **0.035** |
| EFΔ40% | -0.554 | 0.061 | -0.051 | 0.821 |
| EFΔ60% | -0.781 | **0.002** | -0.260 | 0.230 |
| MVPA (hours/week) | -0.135 | 0.405 | -0.377 | **0.013** |
| VPA (hours/week) | -0.287 | 0.072 | -0.224 | 0.149 |
| **Correlates of HRR_2_** |  |  |  |  |
| FEV_1_/FVC (%) | 0.092 | 0.578 | 0.007 | 0.961 |
| % of predicted FVC | -0.444 | **0.005** | -0.148 | 0.327 |
| Vent. Reserve | 0.251 | 0.123 | 0.348 | **0.018** |
| EFΔ40% | -0.537 | 0.072 | 0.087 | 0.699 |
| EFΔ60% | -0.814 | **0.001** | 0.051 | 0.816 |
| MVPA (hours/week) | -0.245 | 0.133 | -0.386 | **0.011** |
| VPA (hours/week) | -0.246 | 0.131 | -0.381 | **0.012** |

FEV_1_ represents forced expiratory volume in 1 s; FVC, forced vital capacity; MVPA, moderate to vigorous physical activity; VPA, vigorous physical activity; EFΔ40%, ejection fraction change from rest to 40% exercise intensity; EFΔ60%, ejection fraction change from rest to 60% exercise intensity.

**REFERENCES**

1. Doherty A, Jackson D, Hammerla N et al. Large Scale Population Assessment of Physical Activity Using Wrist Worn Accelerometers: The UK Biobank Study. PLoS One 2017;12:e0169649.
